# The zoonotic potential of *Clostridium difficile* from small companion animals and their owners

**DOI:** 10.1371/journal.pone.0193411

**Published:** 2018-02-23

**Authors:** Denise Rabold, Werner Espelage, Muna Abu Sin, Tim Eckmanns, Alexander Schneeberg, Heinrich Neubauer, Nadine Möbius, Katja Hille, Lothar H. Wieler, Christian Seyboldt, Antina Lübke-Becker

**Affiliations:** 1 Institute of Microbiology and Epizootics, Department of Veterinary Medicine, Freie Universität Berlin, Berlin, Germany; 2 Robert Koch Institute, Berlin, Germany; 3 Institute of Bacterial Infections and Zoonoses, Federal Research Institute for Animal Health (Friedrich-Loeffler-Institut), Jena, Germany; 4 Department of Biometry, Epidemiology and Information Processing, University of Veterinary Medicine Hannover, Foundation, Hannover, Germany; Institut Pasteur, FRANCE

## Abstract

**Background:**

*Clostridium difficile* infections (CDI) in humans range from asymptomatic carriage to life-threatening intestinal disease. Findings on *C*. *difficile* in various animal species and an overlap in ribotypes (RTs) suggest potential zoonotic transmission. However, the impact of animals for human CDI remains unclear.

**Methods:**

In a large-scale survey we collected 1,447 fecal samples to determine the occurrence of *C*. *difficile* in small companion animals (dogs and cats) and their owners and to assess potential epidemiological links within the community. The Germany-wide survey was conducted from July 2012-August 2013. PCR ribotyping, Multilocus VNTR Analysis (MLVA) and PCR detection of toxin genes were used to characterize isolated *C*. *difficile* strains. A database was defined and logistic regression used to identify putative factors associated with fecal shedding of *C*. *difficile*.

**Results:**

In total, 1,418 samples met the inclusion criteria. The isolation rates for small companion animals and their owners within the community were similarly low with 3.0% (25/840) and 2.9% (17/578), respectively. PCR ribotyping revealed eight and twelve different RTs in animals and humans, respectively, whereas three RTs were isolated in both, humans and animals. RT 014/0, a well-known human hospital-associated lineage, was predominantly detected in animal samples. Moreover, the potentially highly pathogenic RTs 027 and 078 were isolated from dogs. Even though, *C*. *difficile* did not occur simultaneously in animals and humans sharing the same household. The results of the epidemiological analysis of factors associated with fecal shedding of *C*. *difficile* support the hypothesis of a zoonotic potential.

**Conclusions:**

Molecular characterization and epidemiological analysis revealed that the zoonotic risk for *C*. *difficile* associated with dogs and cats within the community is low but cannot be excluded.

## Introduction

*Clostridium difficile* is the major cause of antibiotic and hospital-associated diarrhea in humans. Since 2001, changes in incidence and epidemiology have fostered discussions about the source of infection and possible transmission routes. Although *C*. *difficile* infections (CDI) are mainly diagnosed in health-care settings, about one quarter of the CDI-cases is estimated to occur within the community [1]. Moreover, the epidemiological link between the majority of symptomatic patients suffering from CDI and a subsequent CDI-patient is still missing, thus, suggesting a community acquisition [2]. In particular, with regard to community-acquired CDI, the overlap of *C*. *difficile* strains isolated from humans and animals has increasingly urged one to explore the significance of *C*. *difficile* isolation in various animal species and its potential for zoonotic transmission [3, 4]. RT 014/0 has been reported to be the most common cause of (CDI-) diarrhea in humans in Europe [5, 6]. Although, RT 014/0 is seldom involved in severe epidemic outbreaks, it seems to have particular adaptive capabilities since it can be found in a broad spectrum of animal species [7, 8]. The third most prevalent RT in humans in European countries is RT 078 [5] which is also the most common RT in bovine and porcine populations [9, 10]. Identical isolates from human and livestock samples and a genetic relatedness between human and porcine RT 078 strains have been described before [11–13]. In addition, identical RT 078 strains shared by farmers and their pigs have also been identified [14]. These findings have triggered concerns about the zoonotic transmission of this important pathogen. Moreover, the emergence of RT 027 has been particularly linked to elevated rates of CDI in humans in Europe and Northern America [15, 16]. Interestingly, RT 027 has also been previously isolated from cattle and horses [7] for example, though data about RT 027 in companion animals are rare.

Recent reports of *C*. *difficile* colonization and infection in dogs indicate that *C*. *difficile* has also potentially emerged as a pathogen of small companion animals [17, 18]. However, epidemiological data concerning companion animals are scarce. In Germany, surveys addressing the occurrence of *C*. *difficile* in dogs and cats are restricted to an investigation in animal shelters [19] and the reports about dogs and cats originating from veterinary clinics published nearly 30 years ago [20, 21].

While age, hospitalization and prior antibiotic exposure are confirmed risk factors for CDI in humans [22], factors associated with the isolation of *C*. *difficile* in small companion animals (dogs and cats) are widely unknown. Therefore, we aimed to comparatively determine the isolation rates for *C*. *difficile* in dogs, cats and their owners, to describe the molecular characteristics of the isolates and analyze the putative impact of demographic factors and variables such as health status, prior medication, diet/feeding, and intensity of contact between humans and animals on the occurrence of *C*. *difficile*.

## Materials and methods

Convenience samples from dog- and/or cat-owning households were acquired between July 2012 and August 2013, through personal contacts, kennel clubs, veterinarians, advertisements in professional journals and social media. To participate in the study, at least one dog or cat and at least one person (animal owner), living in the same household had to meet the inclusion criteria which were residency in Germany, signed consent forms, filled in questionnaires and one fecal sample per participating household member. For each participating household one person was defined as a contact person and was personally instructed about the study (by proxy for other participating household members if applicable), and was sent a package containing individually labeled stool containers for self-sampling, information and instruction sheets, consent forms, as well as a self-reporting questionnaire for each participating household member. All samples originating from the same household were collected on the same day. Prior to sampling, the study protocol was approved by the Ethical Committee of the Charité, Campus Virchow-Clinic Berlin.

### Questionnaires

The questionnaire (S1 and S2 Figs) for animal participants covered demographic factors such as breed, age, sex, and whether the animal was neutered as well as details of husbandry (keeping inside/free roaming), and stay in different sites (e.g., sanctuary, animal shows). The questionnaire for animal owners requested demographic data like age, gender, profession, residence and residential environment (e.g., city, countryside). Additional questions assessed the presence of other household members such as children and individuals with chronic disease, confirmed CDI or persons who underwent chemotherapy. For both, animals and their owners, additional information was collected about contact to other animals, intensity of contact between the participating animal and owner (e.g., frequency and type of dog handling, including physical contact), food/feed consumption, status of health (e.g. diarrhea in previous months), prior hospitalization, intake of medication (e.g. antibiotics), and contact to other individuals suffering from diarrhea or with a recent hospital stay.

### Statistical analysis

Statistical analysis was performed using the software STATA® (StataCorp. 2013. Release 13. College Station, TX: StataCorp LP). Univariate analysis examined the association (odds ratio) between potential exposure and outcome using logistic regression with dichotomous and categorial independent variables at significance level of *p* ≤ 0.05. Odds ratios (OR) were calculated with a 95% confidence interval (CI).

Sensitivity analyses were obtained to detect potential clustering. For this, (1) only households participating with one animal and one pet owner were included, (2) all households were considered, though, only one data set (ratio animal-pet owner 1:1) was included, (3) all households were considered, though, only data sets with the ratio animal-pet owner n:n were included, (4) all households were included with complete data sets (ratio animal-pet owner n:m). No significant efficiency differences or increase in performance compared to the univariate analysis of the whole data set were detected; thus, model (4) was selected for the statistical analysis.

For the multivariate analysis variables with *p* ≤ 0.2 associated with isolation of *C*. *difficile* from the univariate analysis were considered as potential risk factors. To select the variables which entered in the final multivariate logistic model a stepwise backward removal procedure with a threshold *p*-value 0.05 was used as implemented in STATA.

### Isolation and molecular characterization of *C*. *difficile*

For *C*. *difficile* cultivation, isolation and identification 2–3 g of each fecal sample was inoculated in 10 ml *C*. *difficile* moxalactam-norfloxacin broth (CDMN, SR173, Oxoid Ltd., Hampshire, United Kingdom) and then underwent direct plating and enrichment culturing as described by Schneeberg et al. [10]. Genomic DNA extraction, toxin gene detection, seq-PCR ribotyping, and Multilocus Variable Number of Tandem Repeat Analysis (MLVA) were performed as previously reported by Schneeberg et al. [10, 23].

We declare that we comply with the terms of service for the websites from which we collected data.

## Results

### *C*. *difficile* isolation rates

In total, 415 households were included in the study; these households were geographically distributed throughout Germany with participants from all 16 federal states. Twelve households did not meet the inclusion criteria for the following reasons: missing questionnaires and/or fecal sample(s) of participating household members, incomplete consent forms, or mixed up ID numbers by different household members. Almost half of the households (46%; 190/415) contributed one animal and one human fecal sample with corresponding questionnaires. Other participant compositions from the same household were manifold, with two animal participants and one owner (11%; 45), one animal and two owners (10%; 40), and two animals and two owners (9%; 36) being the most common.

In total, 1,447 fecal samples were collected, of which 29 samples did not meet the inclusion criteria either due to the provision of more than one sample from the same participant, or the above mentioned reasons. A total of 1,418 fecal samples with 59.2% (840) of animal and 40.8% (578) of human origin were included. *C*. *difficile* was isolated from 42/1418 (3.0%) fecal samples with isolation rates of 3.0% (25/840) for animal and 2.9% (17/578) for human samples (Table 1).

**Table 1:** *Clostridium difficile* isolates in a Germany-wide survey, July 2012-August 2013.

	in total	human	animal	dogs	cats		
Considered samples	1,418	578	840	437	403		
Positive samples (%)	42 (3.0)	17 (2.9)	25 (3.0)	15 (3.4)	10 (2.5)		
*C*. *difficile* isolates	44	18*^h^	26*^a^	16*^a^	10		
Different ribotypes	17	12	8	6	5		

*^h^: two different RTs were isolated in sample 0748M2

*^a^: two different RTs were isolated in sample 0934T1

*Authors’ comment*: three RTs were isolated in humans and animals but did not originate from the same household

### Characterization of *C*. *difficile* isolates

Animal *C*. *difficile* isolates were assigned to eight different PCR ribotypes (RTs) (001/5/FLI01, 009, 010, 014/0, 014/0/FLI01, 027, 039, 078) (Table 2). From one canine sample two *C*. *difficile* strains corresponding to different RTs were isolated (RTs 010 and 039). The predominant RT was RT 014/0, which was determined for 10/26 (38.5%) isolates. RT 010 was found in 5/26 (19.2%) isolates, whereas RTs 001/5/FLI01 and 039 were each detected in 3/26 (11.5%) samples. RTs 014/0/FLI01, 027 and 078 were each isolated from 1/26 (3.8%) isolates. RTs 027 and 078 are often described as highly pathogenic for humans, here they originated from canine participants.

**Table 2:** PCR-Ribotypes and toxin gene detection in *Clostridium difficile* isolates.

	Toxin Genes				Sample Origin			
PCR-Ribotype	*tcdA*	*tcdB*	*cdtA*	*cdtB*	human	dog	cat	
								in total
001/5/FLI01	+	+	-	-	0	0	3	**3**
003	+	+	-	-	2	0	0	**2**
003/FLI02	+	+	-	-	1	0	0	**1**
009	-	-	-	-	0	2	0	**2**
009/FLI01	-	-	-	-	1	0	0	**1**
**010**	-	-	-	-	1	4	1	**6**
010/FLI01	-	-	-	-	1	0	0	**1**
**014/0**	+	+	-	-	4	6	4	**14**
014/0/FLI01	+	+	-	-	0	0	1	**1**
014/5	+	+	-	-	1	0	0	**1**
020	+	+	-	-	1	0	0	**1**
**027**	+	+	+	+	0	1	0	**1**
039	-	-	-	-	0	2	1	**3**
070	+	+	-	-	1	0	0	**1**
**078**	+	+	+	+	3	1	0	**4**
087	+	+	-	-	1	0	0	**1**
441/FLI01	-	-	-	-	1	0	0	**1**
**total**					**18**	**16**	**10**	**44**

Within two households identical RTs were isolated from two animals (in both cases cats which harbored RT 014/0). Further characterization applying MLVA proved that the strains isolated within the same household were clonally related, with a STDR ≤ 2 (Table 3). (The complete results of MLVA-analysis of all isolated strains are available in S1 Table.)

**Table 3:** *Clostridium difficile* isolates in four cats from two independent households.

Partici-pant ID	PCR-RT	MLVA							STRD	Toxin genes				
		A6_*Cd*_	B7_*Cd*_	F3_*Cd*_	H9_*Cd*_	G8_*Cd*_	E7_*Cd*_	C6_*Cd*_		*tcdA*	*tcdB*	*cdtA*	*cdtB*	*cdd3*
0770T2	014/0	28	14	4	2	7	6	9		1	1	0	0	1
0770T4	014/0	28	14	4	2	7	6	9	0	1	1	0	0	1
0919T2	014/0	32	20	4	2	8	6	31		1	1	0	0	1
0919T3	014/0	32	19	4	2	8	6	30	2	1	1	0	0	1

ID: identification number; PCR-RT: PCR-ribotype; MLVA: Multilocus Variable Number of Tandem Repeat Analysis; STRD: Summed Tandem-Repeat Differences, STRD relating to the previous isolate; *tcdA*: gene encoding for toxin A; *tcdB*: gene encoding for toxin B; *cdtA* and *cdtB*: genes encoding for the binary toxin called CDT; *cdd3*: gene encoding an ABC-type transport system, *cdd3*-PCR performed as a species proof.

Human *C*. *difficile* isolates belonged to 12 different RTs (003, 003/FLI02, 009/FLI01, 010, 010/FLI01, 014/0, 014/5, 020, 070, 078, 087, 441/FLI01). In one human sample two different RTs (RTs 003 and 078) were detected. The most prevalent human RT was RT 014/0, which was ascertained in 22.2% (4/18) of the human isolates. RT 078 was isolated from 3/18 (16.7%) and RT 003 from 2/18 (11.1%) isolates. RTs 003/FLI02, 009/FLI01, 010, 010/FLI01, 014/5, 020, 070, 087 and 441/FLI01 were each determined in 1/18 (5.6%) isolates. No parallel occurrence of *C*. *difficile* in dogs or cats and their owners sharing the same household was detected.

In total, 61.5% (16/26) of the animal isolates were positive for toxin genes. Of those, 87.5% (14/16) were *tcdA-* and *tcdB*-positive (genes encoding for toxins A and B) with an additional 12.5% (2/16) also yielding positive PCR results for genes *cdtA* and *cdtB* encoding for the binary toxin (Table 2). In comparison, 77.7% (14/18) of the human isolates were toxigenic. Of these, 78.6% (11/14) were *tcdA-* and *tcdB*-positive with an additional 21.4% (3/14) positive for *cdtA*- and *cdtB*-genes. In contrast, non-toxigenic RTs accounted for 38.5% (10/26) and 22.2% (4/18) in animal and human samples, respectively.

### Statistical analysis

Significant factors associated with *C*. *difficile*-positivity are listed in Tables 4 and 5. (For the complete univariate analysis see S2 and S3 Tables.)

**Table 4:** Univariate analysis for fecal shedding of *C*. *difficile* in dogs and cats (Extraction).

	CD positive	CD negative	*p*-Value	OR	95% CI
**Demographic factors**					
**Age in years**					
< 1	1	44	0.651	1.65	0.19–14.45
1–4	5	363	Ref.	.	.
5–9	13	267	0.018	3.53	1.25–10.04
10–22	6	141	0.066	3.09	0.93–10.28
**Health status**					
Inappetence	5 (20)	28 (750)	<0.001	6.70	2.34–19.14
Acute disease	7 (16)	53 (746)	<0.001	6.16	2.43–15.62
Diarrhea during the last 4 weeks	8 (15)	127 (650)	0.025	2.73	1.13–6.57
**Medication**					
Anti-inflammatory drugs (regular intake)	3 (21)	34 (772)	0.067	3.24	0.92–11.41
Proton pump inhibitors (regular intake)	4 (21)	6 (801)	<0.001	25.43	6.68–96.85
Antibiotics (during the preceding 3 months)	12 (13)	141 (667)	<0.001	4.37	1.95–9.77
**Food consumption**					
Dry food	14 (11)	741 (74)	<0.001	0.13	0.06–0.29
Dog/cat treats	15 (10)	592 (223)	0.170	0.57	0.25–1.28
**Contacts—human/ animal**					
Contact to a human with diarrhea	11 (6)	214 (379)	0.022	3.25	1.18–8.90
Owner of the tested animal has suffered from diarrhea during the last 4 weeks	6 (14)	86 (399)	0.171	1.99	0.74–5.32
Owner of the tested pet suffers from chronic disease	10 (9)	113 (370)	0.006	3.64	1.44–9.17
Person with chronic disease lives in the same household	9 (9)	135 (332)	0.062	2.46	0.96–6.33
**Intensity of contact between owner and animal**					
The animal is allowed to…					
… sleep in bed	18 (7)	521 (285)	0.450	1.41	0.58–3.41
… be washed in the tub/shower	16 (9)	352 (443)	0.057	2.24	0.98–5.12
… lick the owner’s face	18 (7)	471 (319)	0.219	1.74	0.72–4.22

CD: *Clostridium difficile* isolation; Ref.: reference category; OR: odds ratio; CI: confidence interval. *Authors’ comment*: bracketed data indicate the number of participants not applying to the variable in row.

**Table 5:** Univariate analysis for fecal shedding of *C*. *difficile* in animal owners (Extraction).

	CD positive	CD negative	*p*-Value	OR	95% CI
**Demographic factors**					
**Age in years**					
<1	2	1	0.001	70.00	5.41–905.30
1–4	1	4	0.072	8.75	0.82–93.11
5–17	0	26	.	.	.
18–44	5	175	Ref.		
45–64	7	274	0.850	0.89	0.28–2.86
65–87	2	69	0.986	1.01	0.19–5.35
**Profession/ field of occupation**					
Health care	1 (16)	101 (460)	0.225	0.28	0.04–2.17
Other field of action*	4 (13)	293 (268)	0.028	0.28	0.09–0.87
No current occupation (e.g. retirement, parental leave)	9 (8)	138 (423)	0.013	3.45	1.31–9.11
**Health status**					
Chronic disease	6 (10)	134 (423)	0.224	1.89	0.68–5.31
Diarrhea during the last 4 weeks	3 (14)	106 (451)	0.886	0.91	0.26–3.23
**Medication**					
Anti-inflammatory drugs (regular intake)	3 (14)	81 (475)	0.724	1.26	0.35–4.47
Proton pump inhibitors (regular intake)	2 (15)	49 (505)	0.679	1.37	0.31–6.18
Antibiotics (during the preceding 3 months)	9 (8)	70 (485)	<0.001	7.79	2.91–20.87

CD: *Clostridium difficile* isolation; Ref.: reference category; OR: odds ratio; CI: confidence interval

*: excluding professions in agriculture, food production and health care. *Authors’ comment*: bracketed data indicate the number of participants not applying to the variable in row.

In the multivariate analysis we included all animal variables with a *p* ≤ 0.2 in the univariate analysis (Table 6). The regular intake of proton pump inhibitors (PPI) (OR 14.82, 95% CI 1.73–126.78, *p* = 0.014), antibiotic treatment during the preceding 3 months (OR 4.13; 95% CI 1.44–11.84, *p* = 0.008), contact to a human with diarrhea (OR 2.94; 95% CI 1.01–8.60, *p* = 0.048), and the consumption of dry food (OR 0.13; 95% CI 0.04–0.42, *p* = 0.001) remained independently associated with the outcome in our model.

**Table 6:** Multivariate analysis for fecal shedding of *C*. *difficile* in dogs and cats.

	OR	95% CI	*p*-Value
Consumption of dry food	0.13	0.04–0.42	0.001
Regular intake of proton pump inhibitors	14.82	1.73–126.78	0.014
Intake of antibiotics during the preceding 3 months	4.13	1.44–11.84	0.008
Contact to a human with diarrhea	2.94	1.01–8.60	0.048

For human *C*. *difficile* detection the numbers of potential predictor variables with sufficient observations were considered to be too low to produce stable estimates to identify independent risk factors in the logistic regression. Therefore, we present the results of the univariate analysis only.

## Discussion

This is the first systematic large-scale survey presenting data on the occurrence, molecular characteristics and potential risk factors of *C*. *difficile* in small companion animals and their corresponding owners within the community. Since *C*. *difficile* isolation rates in dogs and cats have been reported to range between 1 and 58% [24, 25], the isolation rate observed within this study (3.0% (25/840)) is low despite of sensitive detection methods [3, 26]. However, comparison is difficult as there is no uniform approach of study designs and populations [19]. For example, most of the former studies focused on animal populations in veterinary settings or shelters. There are hardly any data on *C*. *difficile* colonization rates from asymptomatic non-hospitalized humans [27], and pet owners. Nonetheless, the results of our study are in line with an estimated 3% asymptomatic carriage in humans within the community [28], and even somewhat higher than in a recent cross-sectional population-based study conducted in the Netherlands reporting a prevalence rate of 1.23% [29].

Among the 17 different isolated ribotypes RT 014/0 was the most prevalent in this survey, detected in 38.5% of animal and 19.2% of human isolates. Interestingly, in two independent households, four cats (two in each household) carried RT 014/0. The strains differed between the households, whereas within the households the partner cats harbored clonally related 014/0 strains as observed through MLVA. This indicates that transmission between individuals in the same household is possible and confirms the supposed considerable endemic potential of RT 014/0 [6, 7]. However, whether a common source of infection or intra- and/or interspecies transmission cause widespread dissemination of RT 014/0 remains unclear.

*C*. *difficile* RT 078, often discussed as a highly human pathogenic, hypervirulent strain, was also detected in human and animal samples in this study. The detection of RT 078 in one canine and three human samples in our study indicates that not just livestock but companion animals might also play a role in interspecies transmission of this RT.

Moreover, here we report on the first RT 027-isolation from a dog outside Canada as this human epidemic RT has, so far, only been described by Lefebvre et al. in two healthy hospital visitation dogs [30, 31]. This shows that epidemic RTs primarily associated with humans can also occur in companion animals raising the question whether animals can serve as a reservoir for humans or contrarily, whether humans rather pose an infectious risk for animals. Nevertheless, whole genome data are needed to investigate the phylogeny of human and companion animal isolates.

We did not detect *C*. *difficile* in dogs or cats and their owners sharing the same household, simultaneously. Yet, the overall low isolation rate within the community will have impaired the chances of detecting isolate pairs. In a smaller scale survey examining the environment of eight households of recurrent CDI-patients [32], none of the eight tested pets were positive. However, the overlap in human and animal RTs has been described thoroughly and the genetic relatedness of certain strains suggests that interspecies transmission as well as zoonotic transmission is probably possible to occur [3, 6]. Recently, Loo et al. [33] detected *C*. *difficile* in dogs and cats in households of CDI-patients (index cases). Although the authors presented indistinguishable *C*. *difficile*-profiles by pulsed-field gel electrophoresis (PFGE) originating from animal-human isolate pairs, the discriminatory power of PFGE does not enable a full molecular characterization of strains. Hence, zoonotic transmission of *C*. *difficile* remains to be verified. Although the study populations described by Shaughnessy et al. [32] and Loo et al. [33] consisted of human participants with a history of CDI in contrast to the participants in this survey, here, we confirmed that clinically relevant RTs are shared between mainly asymptomatic human carriers and small companion animals. Moreover, the high proportion of toxigenic strains raises concerns that interspecies transmission would have a clinical impact.

In our statistical analysis we identified common risk factors associated with human CDI for dogs and cats as well. Antibiotic treatment has been recognized as the major risk factor for CDI [34]. The disruption of healthy microbiota by antibiotics is indisputable, thus, allowing *C*. *difficile* to cause disease; this is also applicable in human and animal participants in this survey. In contrast, whether the use of PPI or AID increases the risk for CDI in humans has been discussed controversially [35–38]. In this study, intake of PPI or AID was not a significant risk factor for humans who were *C*. *difficile*-positive. In small companion animals the use of AID may be associated with fecal shedding of *C*. *difficile*, with a *p-*value slightly greater than 0.05. In the study population presented here, we were able to show the significant association of antibiotics and PPI with *C*. *difficile* detection in small companion animals. To our knowledge, we are the first to report the association between PPI treatment and *C*. *difficile*-positivity in dogs and cats. Within our study population, PPI was more often administered to dogs (9/10), suggesting that medicating dogs with PPI severely increases the risk of *C*. *difficile* being present in fecal samples. Additionally, not only certain pharmaceuticals but also inappetence could be linked with *C*. *difficile*-positivity in the univariate analysis in this study. Yet, whether inappetence leads to disruption of the intestinal flora and can thereby enable colonization, infection or transient passage with *C*. *difficile*, or whether inappetence is caused by *C*. *difficile* remains speculative. Here, dogs or cats suffering from inappetence or acute disease were six-fold more often positively tested for *C*. *difficile* in the univariate model; besides, animals suffering from acute disease were often treated with antibiotics.

The significant decrease in the likelihood of animals being *C*. *difficile*-positive when fed with dry food was surprising. This variable was also confirmed in the final model as an independent factor showing that pets fed on dry feedstuffs are approximately 10-times less likely to harbor *C*. *difficile*. A possible explanation could be that high temperatures for desiccation during food processing could impair the survival of vegetative cells and spores of *C*. *difficile*. Commercial dry dog and cat feedstuffs are mainly produced using extrusion processes with temperatures up to 200°C [39, 40]. Although *C*. *difficile* spores are able to survive temperatures of 71°C for at least 120 min [41], the extreme conditions during food processing could prevent survival. Besides the composition of diet, the feeding strategy can also influence the microbiota as dry feedstuffs are often fed *ad libitum* to dogs and cats. The combination of feeding strategy and a probably low contamination of dry feedstuffs seem to have a positive effect on the intestinal microenvironment of companion animals.

Despite the absence of animal-human isolate pairs, the results of the epidemiological analysis of factors associated with fecal shedding of *C*. *difficile* in our univariate analysis support the hypothesis of a zoonotic potential for *C*. *difficile*. In particular, we found that companion animals tend to be *C*. *difficile*-positive more often when (1) the owner suffered from a chronic disease (*p* = 0.006) or, (2) has recently suffered from diarrhea (*p* = 0.171), (3) the animal was in contact with a diarrheic person (*p* = 0.022), (4) washed in the tub/shower (*p* = 0.057) and/or (5) a chronically sick person lived in the same household (*p* = 0.062). The latter risk factor was already previously described as contact to immunocompromised individuals [42]. This indicates that sharing the environment with humans influences whether companion animals harbor *C*. *difficile*. Even in our multivariate analysis, contact to a person with diarrhea was found to be an independent risk factor, increasing the chances for dogs and cats of becoming colonized or infected with *C*. *difficile* three-fold. This observation supports the findings by Lefebvre et al. [30, 31] describing hospital visiting dogs acquiring *C*. *difficile* in health-care facilities. Thus, speculations of the impact of animals for human CDI should probably be reassessed as humans might rather pose a risk for animals than the other way around. Nonetheless, they might be a source for reinfection.

One limitation of the study is that sampling was restricted to one fecal sample per individual. Repeated sampling could have increased isolation rates and would have increased the chance to detect intermittent shedding, and thereby, finding animal-human isolate pairs.

In conclusion, well known human ribotypes like RT 010, the hospital-associated lineage RT 014/0 and the potentially virulent RTs 027 and 078 also occur in cats and dogs suggesting at least a common source of infection. Moreover, previously described risk factors for *C*. *difficile* colonization or infection in humans also apply in companion animals. To define possible sources of *C*. *difficile* acquisition and to clarify its zoonotic character further studies involving humans and animals are required.

## Supporting information

S1 Fig *C*.*difficile*-questionnaire in German. (PDF)

S2 Fig *C*.*difficile*-questionnaire in English. (PDF)

S1 Table Complete characterization by means of MLVA for all *C*. *difficile* isolates. (DOCX)

S2 Table Complete univariate analysis for fecal shedding of *C*. *difficile* in dogs and cats. (DOCX)

S3 Table Complete univariate analysis for fecal shedding of *C*. *difficile* in animal owners. (DOCX)
