# Supplementary material for: The zoonotic potential of Clostridium difficile from small companion animals and their owners
Source: PLoS One. 2018 Feb 23;13(2):e0193411. doi: 10.1371/journal.pone.0193411 (PMC5825086; doi:10.1371/journal.pone.0193411)
Supplement: S1 Fig — (PDF) [file pone.0193411.s001.pdf]

CDI.zoo — Freie Universität Berlin — Friedrich-Loeffler-Institut FLI, Bundesforschungsinstitut für Tiergesundheit, Federal Research Institute for Animal Health — Robert Koch Institut

Untersuchungen zur Prävalenz und Typisierung von *Clostridium difficile* bei Haustieren und ihren Haltern

I. Angaben zur Tierhaltung allgemein

1. Halten Sie außer dem heute getesteten Tier weitere Nutz- oder Haustiere?
☐ Ja ☐ Nein ☐ Keine Angabe

↳ falls ja, welche Tiere (Mehrfachnennungen möglich)? Bitte geben Sie die Anzahl an: ....

..... Hunde ..... Katzen ..... Schafe ..... Geflügel ..... Wildtiere ..... Kleinsäuger
..... Pferde ..... Rinder ..... Schweine ..... andere (Tierart bitte angeben): ....

2. Hatten Sie in den letzten 12 Monaten mehrfach Kontakt zu anderen Tieren, deren Besitzer nicht Sie sind? (z.B. Haustiere, Jagd...)?
☐ Ja ☐ Nein ☐ Keine Angabe

↳ falls ja, welche Tiere (Mehrfachnennungen möglich)

☐ Hunde ☐ Katzen ☐ Schafe ☐ Geflügel ☐ Wildtiere ☐ Kleinsäuger
☐ Pferde ☐ Rinder ☐ Schweine ☐ andere (bitte angeben): ....

Aufkleber
Tier ID

II. Angaben zum getesteten Tier

3. Bei dem getesteten Tier handelt es sich um: ☐ Hund ☐ Katze

4. Rasse des Tieres?	5. Geschlecht des Tieres?	6. Kastriert?	7. Alter:
....	☐ weiblich<br>☐ männlich	☐ Ja<br>☐ Nein	.... Jahre

8a) *(falls Hund)* Wie wird dieser Hund hauptsächlich (mehr als die Hälfte der Zeit) gehalten?
☐ im Haus/in der Wohnung
☐ im Zwinger/Garten
☐ im separaten Nebengebäude (z.B. Stall)

8b) *(falls Katze)* Wie wird diese Katze gehalten?
☐ im Haus/in der Wohnung
☐ Freigänger

↳ *falls Freigänger*, wie häufig befindet sich Ihre Katze im Freien?

☐ täglich: .... Stunden pro Tag.
☐ mehrmals in der Woche: .... Stunden pro Woche

9. Hat Ihr Tier regelmäßigen Kontakt zu anderen Nutz- oder Haustieren?
☐ Ja ☐ Nein ☐ Keine Angabe

↳ *falls ja*, zu welchen Tieren (Mehrfachnennungen möglich)?

☐ Hunde ☐ Katzen ☐ Schafe ☐ Geflügel ☐ Wildtiere ☐ Kleinsäuger
☐ Pferde ☐ Rinder ☐ Schweine ☐ andere (bitte angeben): ....

10. Hatte Ihr Tier in den letzten 12 Monaten regelmäßigen Kontakt zu Jungtieren (z.B. Welpen, Ferkeln, ...)?
☐ Ja ☐ Nein ☐ Keine Angabe

↳ *falls ja*, zu welchen Tieren (Mehrfachnennungen möglich)?

☐ Hunde ☐ Katzen ☐ Schafe ☐ Geflügel ☐ Wildtiere ☐ Kleinsäuger
☐ Pferde ☐ Rinder ☐ Schweine ☐ andere (bitte angeben): ....

11. Welche Aussage ist zutreffend, wenn Sie für die letzten 12 Monate den Kontakt zu Ihrem Tier beschreiben?

Ihr Tier...	Täglich	Mehrmals in der Woche	Mehrmals im Monat	Selten	Nie
a) ... darf auf dem Sofa liegen	☐	☐	☐	☐	☐
b) ... darf mit in Ihr Bett	☐	☐	☐	☐	☐
c) ... wird in Ihrer Badewanne/Dusche gewaschen	☐	☐	☐	☐	☐
d) ... wird von Ihnen gestreichelt/gekrault	☐	☐	☐	☐	☐
e) ... darf aus der Hand fressen	☐	☐	☐	☐	☐
f) ... darf Ihr Gesicht lecken	☐	☐	☐	☐	☐
g) Sonstiger Kontakt zu dem Tier	☐	☐	☐	☐	

(bitte angeben): ....

... weitere Angaben zum getesteten Tier...

12. Hat sich Ihr Tier innerhalb der letzten 12 Monate in einer der folgenden Einrichtungen aufgehalten?

a) Tierpension	☐ Ja: Wann zuletzt (Monat/Jahr)? ...../........	☐ Nein
b) Tierheim	☐ Ja: Wann zuletzt (Monat/Jahr)? ...../........	☐ Nein
c) Kindertagesstätte, Schule	☐ Ja: Wann zuletzt (Monat/Jahr)? ...../........	☐ Nein
d) Tierschau/Ausstellung	☐ Ja: Wann zuletzt (Monat/Jahr)? ...../........	☐ Nein
e) Pflege-Einrichtung / Reha-Einrichtung	☐ Ja: Wann zuletzt (Monat/Jahr)? ...../........	☐ Nein
f) als Therapie-Tier eingesetzt (z.B. im Krankenhaus)	☐ Ja: Wann zuletzt (Monat/Jahr)? ...../........	☐ Nein
g) Hundeschule / Hundesport	☐ Ja: Wann zuletzt (Monat/Jahr)? ...../........	☐ Nein

13. Welches der folgenden Futtermittel frisst Ihr Tier? (Mehrfachantworten möglich)
- ☐ Nassfutter aus Dose oder Beutel
- ☐ Trockenfutter
- ☐ Trockenfleischprodukte (z.B. Schweineohr)
- ☐ Frischfleisch, roh (z.B. Pansen etc.)
- ☐ Speisereste/Tischabfälle
- ☐ Leckerlies (z.B. Kauknochen, Katzensticks etc.)
- ☐ Ergänzungsfuttermittel (z.B. Vitamin- oder Mineralstoffpräparate)
- ☐ sonstiges (bitte angeben): ....

14. Neigt Ihr Tier zu...?

a) Fressen von Kot (Koprophagie)	☐ Ja	☐ Nein	☐ unbekannt
b) Gefräßigkeit, z.B. Aufnahme von Müll (Polyphagie)	☐ Ja	☐ Nein	☐ unbekannt
c) Appetitlosigkeit (Inappetenz)	☐ Ja	☐ Nein	☐ unbekannt

15. Leidet Ihr Tier an einer akuten Erkrankung? (kurzer Krankheitsverlauf von 3-14 Tagen)
☐ Ja, und zwar: .... ☐ Nein ☐ unbekannt

16. Leidet Ihr Tier an chronischen Erkrankungen?
(langer Krankheitsverlauf von über 14 Tagen, z.B. Diabetes, Hauterkrankung, Tumor, Epilepsie...)
☐ Ja, und zwar: .... ☐ Nein ☐ unbekannt

17. Muss Ihr Tier regelmäßig entzündungshemmende Medikamente (z.B. Metacam®, Rimadyl®) einnehmen?
☐ Ja ☐ Nein ☐ unbekannt ☐ keine Angabe
↳ *falls ja*, seit wann? (Monat/Jahr)? ..../.............

18. Muss Ihr Tier regelmäßig Magensäure-blockende Medikamente einnehmen?
(sog. Protonenpumpenhemmer, z.B. Omeprazol)
☐ Ja ☐ Nein ☐ unbekannt ☐ keine Angabe
↳ *falls ja*, seit wann? (Monat/Jahr)? ..../.............

19. Wurde Ihr Tier innerhalb der letzten 3 Monate mit Antibiotika behandelt? (außer Wurmkur)
☐ Ja ☐ Nein ☐ unbekannt ☐ Keine Angabe
↳ *falls ja*: Warum? Diagnose/Erkrankung: ....
Wann? (Monat/Jahr)? ..../.............
Wie? ☐ Lokale Behandlung (Salben, Augentropfen)
☐ Systemische Behandlung (Tabletten, Spritzen, o.ä.)

20. Hatte Ihr Tier in den letzten 4 Wochen Durchfall?
(Unter Durchfall verstehen wir mehr als 3 ungeformte Stühle/Tag.)
☐ Ja ☐ Nein ☐ unbekannt ☐ Keine Angabe
↳ *falls ja*, wie lange dauerte der Durchfall an?
☐ wenige Tage bis max. 3 Wochen ☐ länger als 3 Wochen ☐ keine Angabe

21. Hatte Ihr Tier innerhalb der letzten 12 Monate Kontakt zu einem Menschen oder Tier mit Durchfall?
☐ Ja ☐ Nein ☐ unbekannt ☐ Keine Angabe
↳ *falls ja*, ☐ zu einem Menschen. Wann letztmalig? (Monat/Jahr)? ..../.............
☐ zu einem Tier. Wann letztmalig? (Monat/Jahr)? ..../.............

22. Hatte Ihr Tier innerhalb der letzten 12 Monate einen stationären Aufenthalt in einer Tierklinik?
☐ Ja: Wann war der letzte Aufenthalt? (Monat/Jahr)? ..../............. ☐ Nein ☐ unbekannt

23. Hatte Ihr Tier innerhalb der letzten 12 Monate Kontakt zu einem anderen Patienten (Mensch oder Tier) nachdem dieser kurz zuvor in einer Klinik behandelt wurde?
☐ Ja ☐ Nein ☐ unbekannt ☐ Keine Angabe
↳ *falls ja*, ☐ zu einem Menschen. Wann letztmalig? (Monat/Jahr)? ..../.............
☐ zu einem Tier. Wann letztmalig? (Monat/Jahr)? ..../.............

Aufkleber
Person ID

III. Angaben zum Tierhalter / zur Tierhalterin

24. In welchem Bundesland wohnen Sie? ……………………………………………….

25. Wie würden Sie Ihr Wohnumfeld beschreiben?
☐ Großstadt
☐ Kleinstadt
☐ Auf dem Land
☐ Keine Angabe

26. Geburtsdatum (Monat/Jahr)? ………/…………..

27. Geschlecht
☐ weiblich ☐ männlich

28. In welchem Beruf bzw. Tätigkeitsfeld sind Sie aktuell tätig (Mehrfachnennungen möglich)?
☐ Landwirtschaft, und zwar als: ……………………………………………………………………………
☐ Nahrungsmittelproduktion, und zwar als: ……………………………………………………………
☐ Gesundheitswesen, und zwar als: ………………………………………………………………………
☐ anderer Tätigkeitsbereich, und zwar als: ……………………………………………………………
☐ aktuell keine berufliche Tätigkeit (Elternzeit, Rente, etc.)
☐ keine Angabe

29. Leben in Ihrem Haushalt Kinder bis 16 Jahre?
☐ Ja ☐ Nein ☐ keine Angabe
↳ *falls ja*, bitte geben Sie an wie viele Kinder in welcher Altersgruppe sind:
Anzahl der Kinder unter 2 Jahre: ……… Kind/er
Anzahl der Kinder 2 bis 9 Jahre: ……… Kind/er
Anzahl der Kinder 10 bis 16 Jahre: ……… Kind/er

30. Lebt in Ihrem Haushalt eine Person, die an einer chronischen Erkrankung leidet?
☐ Ja ☐ Nein ☐ unbekannt ☐ keine Angabe
↳ *falls ja*, welche Erkrankung? ……………………………………………………………………………………

31. Lebt in Ihrem Haushalt eine Person oder ein Tier, bei der *Clostridium difficile* nachgewiesen wurde?
☐ Ja ☐ Nein ☐ unbekannt ☐ keine Angabe
↳ *falls ja*, ☐ bei einem Menschen. Wann? (Monat/Jahr)? ……/…………..
☐ bei einem Tier. Wann? (Monat/Jahr)? ……./…………..

32. Welche der folgenden Lebensmittel nehmen Sie zu sich?

	Täglich	Mehrmals in der Woche	Mehrmals im Monat	Selten	Nie
… Leitungswasser als Kaltgetränk	☐	☐	☐	☐	☐
… Rohmilch/-produkte	☐	☐	☐	☐	☐
… Rohfleisch/-produkte (z.B. Hackfleisch)	☐	☐	☐	☐	☐
… abgepackte Salate	☐	☐	☐	☐	☐
… probiotische Drinks (z.B. Actimel®)	☐	☐	☐	☐	☐

33. Hatten Sie innerhalb der letzten 12 Monate Kontakt zu einem Menschen oder Tier mit Durchfall?
☐ Ja ☐ Nein ☐ unbekannt ☐ keine Angabe
↳ *falls ja*, ☐ zu einem Menschen. Wann letztmalig? (Monat/Jahr)? ……/…………..
☐ zu einem Tier. Wann letztmalig? (Monat/Jahr)? ……/…………..

34. Hatten Sie innerhalb der letzten 12 Monate einen Klinikaufenthalt von mindestens einer Woche?
☐ Ja: Wann war der letzte Aufenthalt? (Monat/Jahr)? ……/……… ☐ Nein ☐ unbekannt ☐ keine Angabe

35. Hatten Sie Kontakt zu einem anderen Patienten (Mensch oder Tier), nachdem dieser in den letzten 12 Monaten in einer Klinik behandelt wurde?
☐ Ja ☐ Nein ☐ unbekannt ☐ keine Angabe
↳ *falls ja*, ☐ zu einem Menschen. Wann? (Monat/Jahr)? ……../…………..
☐ zu einem Tier. Wann? (Monat/Jahr)? ………/…………..

... weitere Angaben zum Tierhalter / zur Tierhalterin

36. Hatten Sie in den letzten 4 Wochen Durchfall?
(Unter Durchfall verstehen wir mehr als 3 ungeformte Stühle pro Tag.)
☐ Ja ☐ Nein ☐ unbekannt ☐ keine Angabe
↳ *falls ja*, wie lange dauerte der Durchfall an?
☐ wenige Tage bis max. 3 Wochen ☐ länger als 3 Wochen ☐ unbekannt

37. Nehmen Sie regelmäßig entzündungshemmende Medikamente (z.B. Aspirin®, Ibuprofen) ein?
☐ Ja ☐ Nein ☐ unbekannt ☐ keine Angabe
↳ *falls ja*, seit wann? (Monat/Jahr)? ..../.............

38. Nehmen Sie regelmäßig Magensäure-blockende Medikamente ein?
(sog. Protonenpumpenhemmer, z.B. Nexium®, Pantozol®)
☐ Ja ☐ Nein ☐ unbekannt ☐ keine Angabe
↳ *falls ja*, seit wann? (Monat/Jahr)? ..../.............

39. Hatten Sie innerhalb der letzten 12 Monate Kontakt zu einem Durchfall-Patienten (Mensch oder Tier)?
☐ Ja ☐ Nein ☐ unbekannt ☐ keine Angabe
↳ *falls ja*, ☐ zu einem Menschen. Wann letztmalig? (Monat/Jahr)? ..../.............
☐ zu einem Tier. Wann letztmalig? (Monat/Jahr)? ..../.............

40. Wurden Sie innerhalb der letzten 2 Monate mit Antibiotika behandelt?
☐ Ja ☐ Nein ☐ unbekannt ☐ Keine Angabe
↳ *falls ja:* Warum? Diagnose/Erkrankung: ....
Wann? (Monat/Jahr)? ..../ ....
Wie? ☐ Lokale Behandlung (Salben, Augentropfen)
☐ Systemische Behandlung (Tabletten, Spritzen, o.ä.)

41. Wurden Sie innerhalb des letzten Jahres mit einer Chemotherapie behandelt?
☐ Ja ☐ Nein ☐ keine Angabe

42. Leiden Sie an einer chronischen Erkrankung (z.B. Diabetes, Neurodermitis, u.a.)?
☐ Ja ☐ Nein ☐ keine Angabe
↳ *falls ja*, welche Erkrankung? ....

43. Ist bei Ihnen schon einmal *Clostridium difficile* nachgewiesen worden?
☐ Ja ☐ Nein ☐ unbekannt ☐ Keine Angabe
↳ *falls ja*, hatten Sie dabei assoziierte Krankheitssymptome?
☐ Ja, und zwar: .... ☐ Nein ☐ unbekannt ☐ keine Angabe

IV. Sonstiges

44. Dürfen wir Sie bei weiteren Fragen im Rahmen dieser Studie nochmals kontaktieren?
☐ Ja ☐ Nein

Vielen Dank für Ihre Mitarbeit!
