# Supplementary material for: The zoonotic potential of Clostridium difficile from small companion animals and their owners
Source: PLoS One. 2018 Feb 23;13(2):e0193411. doi: 10.1371/journal.pone.0193411 (PMC5825086; doi:10.1371/journal.pone.0193411)
Supplement: S2 Fig — (PDF) [file pone.0193411.s002.pdf]

I. General information about animal contacts

1. Have you got other pets or animals apart from the participating animal?
   ☐ Yes ☐ No ☐ n/a

➯ if applicable, which animals? (check all that apply) – Please, specify the quantity..................................

.....dogs .....cats .....sheep .....poultry .....wild animals .....rabbits and other small mammals

.....horses .....cattle .....pigs .....others (please specify): ....

2. Have you been several times in contact with other animals (of which you are not the owner) during the last 12 months? (e.g. pets, hunt…)?

   ☐ Yes ☐ No ☐ n/a

➯ *if applicable,* which animals? (check all that apply)

☐ dogs ☐ cats ☐ sheep ☐ poultry ☐ wild animals ☐ rabbits and other small mammals
☐ horses ☐ cattle ☐ pigs ☐ others (please specify): ....

II. Information about the tested animal

3. The tested animal is a: ☐ dog ☐ cat

4. Which species does the animal belong to? ....

5. Gender? ☐ female ☐ male

6. Neutered? ☐ Yes ☐ No

7. Age: .... years

8a) *(if tested animal is a dog)* Where is the dog most of the times (more than half of the time)?
☐ in the house/flat
☐ in a kennel/garden
☐ in a separate building (e.g. in a stable)

8b) *(if tested animal is a cat)* Where is the cat most of the times?
☐ in the house/flat
☐ outdoors

➯ *if the tested animal is an outdoor-cat,* how often is it outside?

☐ daily: .... hours per day
☐ several times a week: .... hours per week

9. Is the animal in regular contact with other companion animals or livestock?
   ☐ Yes ☐ No ☐ n/A

➯ *if applicable,* which animals? (check all that apply)

☐ dogs ☐ cats ☐ sheep ☐ poultry ☐ wild animals ☐ small companion animals
☐ horses ☐ cattle ☐ pigs ☐ others (please specify): ....

10. Has the tested animal been in contact with neonates/young animals during the last 12 months (e.g., dog puppies, piglets, …)?

   ☐ Yes ☐ No ☐ n/A

➯ *if applicable,* which animals? (check all that apply)

☐ dogs ☐ cats ☐ sheep ☐ poultry ☐ wild animals ☐ small companion animals
☐ horses ☐ cattle ☐ pigs ☐ others (please specify): ....

11. Which of the following is applicable between owner and animal during the last 12 months?

The animal is …	Daily	Several times per week	Several times per month	Rarely	Never
a) …allowed to lie on the couch	☐	☐	☐	☐	☐
b) …allowed to sleep in the owner's bed	☐	☐	☐	☐	☐
c) …washed in the tub/shower	☐	☐	☐	☐	☐
d) …petted	☐	☐	☐	☐	☐
e) …allowed to feed out of the hand	☐	☐	☐	☐	☐
f) …is allowed to lick the owner's face	☐	☐	☐	☐	☐
g) other contacts	☐	☐	☐	☐	☐

(please specify):…………………………………………………………………………………………

12. Has the tested animal been in any of the following facilities during the last 12 months?

a)	kennels/boarding facilities	☐ Yes: when at last (month/year)? ...../.......	☐ No
b)	animal shelter	☐ Yes: when at last (month/year)? ...../.......	☐ No
c)	kindergarten, school	☐ Yes: when at last (month/year)? ...../.......	☐ No
d)	Dog-/cat-show	☐ Yes: when at last (month/year)? ...../.......	☐ No
e)	Health care/rehabilitation facility	☐ Yes: when at last (month/year)? ...../.......	☐ No
f)	in use as a therapy dog (e.g. in hospital)	☐ Yes: when at last (month/year)? ...../.......	☐ No
g)	pet obedience school	☐ Yes: when at last (month/year)? ...../.......	☐ No

...more information about the tested animal...

13. Which of the following does the tested animal feed on? (check all that apply)

☐ Canned food
☐ Dry food
☐ Jerky (e.g. dried pig ears)
☐ Raw meat (products) (e.g. rumen)
☐ Leftovers
☐ Dog/cat treats (e.g. dog chew bones, cat chew treats)
☐ Nutritional supplements (e.g. vitamin or mineral additives)
☐ Others (please specify): ....

14. Is the tested animal prone to …?

a) Feeding on faeces (coprophagy)	☐ Yes	☐ No	☐ unknown
b) Uncontrolled greedy feeding, e.g. consumption of waste (polyphagy)	☐ Yes	☐ No	☐ unknown
c) loss of appetite (inappetence)	☐ Yes	☐ No	☐ unknown

15. Does the tested animal suffer from an acute disease? (short period of 3-14 days)

☐ Yes, from: .... ☐ No ☐ unknown

16. Does the tested animal suffer from a chronic disease? (long period over 14 days, e.g. diabetes, skin disease, tumor, epilepsy)

☐ Yes, from: .... ☐ No ☐ unknown

17. Does the tested animal regularly take in anti-inflammatory drugs (e.g. Metacam®, Rimadyl®)?

☐ Yes ☐ No ☐ unknown ☐ n/A

↳ ***if applicable*, since when? (month/year)?..../.........................**

18. Does the tested animal regularly take in drugs to reduce gastric acidity (so called proton pump inhibitors, e.g. Omeprazol)?

☐ Yes ☐ No ☐ unknown ☐ n/A

↳ ***if applicable*, since when? (month/year)?..../.........................**

19. Has the tested animal been treated with antibiotics during the last 3 months? (no anthelmintic therapy)

☐ Yes ☐ No ☐ unknown ☐ n/A

↳ ***if applicable:*** Why? Diagnosis/Disease: ....
When? (month/year)?..../...............

How? ☐ local treatment (ointment, eyedrops)
☐ systemic treatment (drugs, syringe)

20. Has the tested animal suffered from diarrhoea during the last 4 weeks? (Diarrhoea as in terms of more than 3 unformed faeces/day)

☐ Yes ☐ No ☐ unknown ☐ n/A

↳ ***if applicable*, how long did the diarrhoea last?**

☐ a few days until max. 3 weeks ☐ longer than 3 weeks ☐ n/A

21. Has the tested animal been in contact with a human or animal suffering from diarrhoea?

☐ Yes ☐ No ☐ unknown ☐ n/A

↳ ***if applicable:*** ☐ to a human. When at last? (month/year)?..../..........
☐ to an animal. When at last? (month/year)?..../..........

22. Has the tested animal been hospitalized in a veterinary clinic during the last 12 months?

☐ Yes: When at last? (month/year)?....../.................... ☐ No ☐ unknown

23. During the last 12 months has the tested animal been in contact with a patient (human or animal) who had been recently hospitalized?

☐ Yes ☐ No ☐ unknown ☐ n/A

↳ *if applicable:* ☐ to a human. When at last? (month/year)?....../..........

☐ to an animal. When at last? (month/year)?....../..........

III. Information about the animal owner

24. In which district of Germany do you live in? ....

25. How would you describe your residential environment?

☐ large city
☐ provincial city
☐ countryside
☐ n/A

26. Date of birth (month/year)? ..../....................................

27. Gender

☐ female ☐ male

28. Which is your current field of occupation (check all that apply)?

☐ in agriculture, as: ....
☐ in food production, as: ....
☐ in health care, as: ....
☐ other field of occupation: ....
☐ currently not occupied (parental leave, retirement, etc.)
☐ n/A

29. Do children younger than 16 years of age live in your household?

☐ Yes ☐ No ☐ unknown

↳ *if applicable,* please specify how many children in the following age groups:

number of children younger than 2 years: .... child/ren
number of children between 2 and 9 years: .... child/ren
number of children between 10 and 16 years: .... child/ren

30. Does a chronically sick person live in your household?

☐ Yes ☐ No ☐ unknown ☐ n/A

↳ *if applicable,* which disease? ....

31. Has a person or animal living in your household previously been tested positive for *Clostridium difficile*?

☐ Yes ☐ No ☐ unknown ☐ n/A

↳ *if applicable:* ☐ a person. When? (month/year)?....../....................

☐ an animal. When? (month/year)?....../....................

32. Which of the following food/drinks do you consume?

		Daily	Several times per week	Several times per month	Rarely	Never
h)	Tap water as a cold drink	☐	☐	☐	☐	☐
i)	Raw milk/-products	☐	☐	☐	☐	☐
j)	Raw meat/-products (e.g. mince)	☐	☐	☐	☐	☐
k)	Ready-to-eat-salads	☐	☐	☐	☐	☐
l)	Probiotics (e.g. Actimel®)	☐	☐	☐	☐	☐

33. Have you been in contact with a human or animal suffering from diarrhoea during the last 12 months?

☐ Yes ☐ No ☐ unknown ☐ n/A

↳ *if applicable:* ☐ with a human. When at last? (month/year)?....../........

☐ with an animal. When at last? (month/year)?....../.......

34. Have you been hospitalized for at least one week during the last 12 months?

☐ Yes: When was the last stay (month/year): ..../.......... ☐ No ☐ unknown ☐ n/A

… more questions regarding the animal owner

35. During the last 12 months have you been in contact with a patient (human or animal) who had been recently hospitalized?

☐ Yes ☐ No ☐ unknown ☐ n/A

↳ *if applicable:* ☐ with a human. When at last? (month/year)? ..../........

☐ with an animal. When at last? (month/year)? ..../........

36. Have you suffered from diarrhoea during the last 4 weeks? (Diarrhoea as in terms of more than 3 unformed faeces/day)

☐ Yes ☐ No ☐ unknown ☐ n/A

↳ *if applicable*, how long did the diarrhoea last?

☐ a few days until max. 3 weeks ☐ longer than 3 weeks ☐ n/A

37. Do you regularly take in anti-inflammatory drugs (e.g. Aspirin®, Ibuprofen)?

☐ Yes ☐ No ☐ unknown ☐ n/A

↳ *if applicable*, since when? (month/year)? ..../...........

38. Do you regularly take in drugs to reduce gastric acidity? (so called proton pump inhibitors (e.g. Nexium®, Pantozol®)

☐ Yes ☐ No ☐ unknown ☐ n/A

↳ *if applicable*, since when? (month/year)? ..../...........

39. Have you been in contact to a diarrhoeic patient (human or animal) during the last 12 months?

☐ Yes ☐ No ☐ unknown ☐ n/A

↳ *if applicable:* ☐ to a human. When at last? (month/year)? ..../..........

☐ to an animal. When at last? (month/year)? ..../..........

40. Have you been treated with antibiotics during the last 2 months?

☐ Yes ☐ No ☐ unknown ☐ n/A

↳ *if applicable:* Why? Diagnosis/Disease: ....
When? (month/year)? ..../..............
How? ☐ local treatment (ointment, eyedrops)
☐ systemic treatment (drugs, syringe)

41. Have you received chemotherapy during the last year?

☐ Yes ☐ No ☐ n/A

42. Do you suffer from a chronic disease (e.g. diabetes, neurodermitis, among others)?

☐ Yes ☐ No ☐ n/A

↳ *if applicable,* which disease? ....

43. Have you previously been positively tested for *Clostridium difficile*?

☐ Yes ☐ No ☐ unknown ☐ n/A

↳ *if applicable*, did you suffer from associated symptoms?

☐ Yes, such as: .... ☐ No ☐ unknown ☐ n/A

IV. Miscellaneous

44. Do you allow us further contact in case additional information is necessary within the survey?

☐ Yes ☐ No
