# Supplementary material for: The zoonotic potential of Clostridium difficile from small companion animals and their owners
Source: PLoS One. 2018 Feb 23;13(2):e0193411. doi: 10.1371/journal.pone.0193411 (PMC5825086; doi:10.1371/journal.pone.0193411)
Supplement: S1 Table — (DOCX) [file pone.0193411.s003.docx]

| **ID** | **PCR-Ribotype** | **A6Cd** | **B7Cd** | **F3Cd** | **H9Cd** | **G8Cd** | **E7Cd** | **C6Cd** |
| --- | --- | --- | --- | --- | --- | --- | --- | --- |
| 0199M1 | 009/FLI01 | 17 | 16 | 4 | 2 | 0 | 8 | 16 |
| 0203M1 | 078 | 9 | 20 | 4 | 2 | 9 | 8 | 33 |
| 0212M1 | 010 | 35 | 16 | 4 | 2 | 9 | 8 | 40 |
| 0416M1 | 070 | 36 | 10 | 5 | 1 | 6 | 6 | 44 |
| 0435M1 | 078 | 0 | 19 | 4 | 2 | 9 | 8 | 31 |
| 0674M1 | 010/FLI01 | 34 | 16 | 4 | 2 | 9 | 6 | 39 |
| 0715M1 | 014/0 | 27 | 13 | 4 | 2 | 7 | 6 | 26 |
| 0718M1 | 014/0 | 26 | 18 | 4 | 2 | 7 | 7 | 33 |
| 0721M2 | 020 | 23 | 12 | 4 | 2 | 12 | 5 | 22 |
| 0748M2 | 003 | 17 | 20 | 4 | 1 | 2 | 9 | 26 |
| 0748M2E | 078 | 0 | 19 | 4 | 2 | 9 | 8 | 31 |
| 0798M1 | 003/FLI02 | 44 | 19 | 5 | 1 | 4 | 13 | 33 |
| 0810M1 | 087 | 28 | 10 | 5 | 1 | 10 | 6 | 48 |
| 0858M1 | 441/FLI01 | 22 | 9 | 4 | 2 | 0 | 8 | 11 |
| 0926M3 | 003 | 16 | 20 | 4 | 1 | 2 | 10 | 47 |
| 0947M2 | 014/5 | 33 | 24 | 4 | 2 | 9 | 4 | 42 |
| 0818M2 | 014/0 | 19 | 18 | 4 | 2 | 7 | 6 | 27 |
| 0456M1 | 014/0 | 24 | 20 | 4 | 2 | 7 | 6 | 45 |
| 0231T1 | 014/0 | 23 | 22 | 4 | 2 | 7 | 6 | 32 |
| 0248T1 | 014/0 | 26 | 19 | 4 | 2 | 6 | 6 | 30 |
| 0269T1 | 010 | 14 | 18 | 4 | 2 | 11 | 8 | 43 |
| 0382T1 | 027 | 27 | 8 | 4 | 2 | 16 | 10 | 30 |
| 0570T2 | 001/5/FLI01 | 15 | 1 | 5 | 2 | 7 | 6 | 31 |
| 0652T1 | 014/0 | 23 | 15 | 4 | 2 | 7 | 6 | 21 |
| 0673T1 | 010 | 37 | 18 | 4 | 2 | 9 | 8 | 36 |
| 0730T1 | 009 | 43 | 20 | 5 | 1 | 11 | 2 | 31 |
| 0762T1 | 014/0 | 27 | 16 | 4 | 2 | 12 | 5 | 40 |
| 0765T2 | 001/5/FLI01 | 15 | 1 | 5 | 2 | 7 | 6 | 31 |
| 0770T2 | 014/0 | 28 | 14 | 4 | 2 | 7 | 6 | 9 |
| 0770T4 | 014/0 | 28 | 14 | 4 | 2 | 7 | 6 | 9 |
| 0773T2 | 009 | 14 | 19 | 5 | 1 | 10 | 2 | 17 |
| 0783T4 | 014/0/FLI01 | 34 | 14 | 4 | 2 | 7 | 6 | 29 |
| 0672T1 | 078 | 0 | 19 | 4 | 2 | 9 | 8 | 31 |
| 0824T1 | 039 | 46 | 21 | 7 | 1 | 1 | 2 | 30 |
| 0829T5 | 001/5/FLI01 | 15 | 1 | 5 | 2 | 7 | 6 | 31 |
| 0831T1 | 014/0 | 32 | 21 | 4 | 2 | 9 | 6 | 24 |
| 0837T3 | 014/0 | 33 | 20 | 4 | 2 | 7 | 6 | 22 |
| 0838T1E | 010 | 22 | 16 | 4 | 2 | 12 | 9 | 26 |
| 0895T1 | 010 | 33 | 18 | 4 | 2 | 9 | 8 | 43 |
| 0919T2 | 014/0 | 32 | 20 | 4 | 2 | 8 | 6 | 31 |
| 0919T3 | 014/0 | 32 | 19 | 4 | 2 | 8 | 6 | 30 |
| 0920T1 | 039 | 34 | 21 | 7 | 1 | 1 | 2 | 31 |
| 0934T1 | 039 | 36 | 19 | 7 | 1 | 1 | 2 | 35 |
| 0934T1E | 010 | 39 | 19 | 4 | 2 | 10 | 7 | 40 |
