# Supplementary material for: The zoonotic potential of Clostridium difficile from small companion animals and their owners
Source: PLoS One. 2018 Feb 23;13(2):e0193411. doi: 10.1371/journal.pone.0193411 (PMC5825086; doi:10.1371/journal.pone.0193411)
Supplement: S2 Table — (DOCX) [file pone.0193411.s004.docx]

**Supplementary Table 2.** Complete univariate analysis for fecal shedding of *C. difficile* in dogs and cats.

|  | CD positive | CD negative | n | *p*-Value | OR | 95% CI |
| --- | --- | --- | --- | --- | --- | --- |
| **Demographic factors and animal housing** | | |  |  |  |  |
| Cat (dog) | 10 (15) | 393 (422) | 840 | 0.420 | 0.72 | 0.32-1.61 |
| Female (male) | 12 (13) | 460 (353) | 838 | 0.396 | 0.71 | 0.32-1.57 |
| Neutered (not neutered) | 13 (12) | 398 (406) | 829 | 0.806 | 1.11 | 0.50-2.45 |
| **Age in years** |  |  | 840 |  |  |  |
| < 1 | 1 | 44 | 45 | 0.651 | 1.65 | 0.19-14.45 |
| 1-4 | 5 | 363 | 368 | Ref. | . | . |
| 5-9 | 13 | 267 | 280 | 0.018 | 3.53 | 1.25-10.04 |
| 10-22 | 6 | 141 | 147 | 0.066 | 3.09 | 0.93-10.28 |
| **Animal housing** | |  |  |  |  |  |
| Dog: house/flat (kennel/garden/  stable) | 14 (1) | 361 (50) | 426 | 0.527 | 1.94 | 0.25-15.06 |
| Cat: house/flat (outdoor) | 6 (4) | 290 (85) | 385 | 0.211 | 0.44 | 0.12-1.59 |
| **Health status/ behavior** | | | | | | |
| Coprophagy | 1 (24) | 118 (675) | 818 | 0.162 | 0.24 | 0.03-1.78 |
| Polyphagia | 2 (23) | 102 (683) | 810 | 0.468 | 0.58 | 0.14-2.51 |
| Inappetence | 5 (20) | 28 (750) | 803 | <0.001 | 6.70 | 2.34-19.14 |
| Acute disease | 7 (16) | 53 (746) | 822 | <0.001 | 6.16 | 2.43-15.62 |
| Chronic disease | 6 (19) | 137 (651) | 813 | 0.395 | 1.50 | 0.59-3.83 |
| Diarrhea during the last 4 weeks | 8 (15) | 127 (650) | 800 | 0.025 | 2.73 | 1.13-6.57 |
| **Medication** | | | | | | |
| Anti-inflammatory drugs | 3 (21) | 34 (772) | 830 | 0.067 | 3.24 | 0.92-11.41 |
| Proton pump inhibitors | 4 (21) | 6 (801) | 832 | <0.001 | 25.43 | 6.68-96.85 |
| Antibiotics | 12 (13) | 141 (667) | 833 | <0.001 | 4.37 | 1.95-9.77 |
| **Food consumption** | | | | | | |
| Canned food | 15 (10) | 573 (242) | 840 | 0.272 | 0.63 | 0.28-1.43 |
| Dry food | 14 (11) | 741 (74) | 840 | <0.001 | 0.13 | 0.06-0.29 |
| Jerky | 7 (18) | 263 (552) | 840 | 0.653 | 0.82 | 0.34-1.98 |
| Raw meat (products) | 9 (16) | 303 (512) | 840 | 0.904 | 0.95 | 0.41-2.18 |
| Leftovers | 5 (20) | 183 (632) | 840 | 0.772 | 0.86 | 0.32-2.33 |
| Dog/cat treats | 15 (10) | 592 (223) | 840 | 0.170 | 0.57 | 0.25-1.28 |
| Animal feed additives | 6 (19) | 250 (565) | 840 | 0.477 | 0.71 | 0.28-1.81 |
| Others | 6 (19) | 202 (613) | 840 | 0.929 | 0.96 | 0.38-2.43 |

| **Stay in different sites during the last 12 months** | | | | | | | | | |
| --- | --- | --- | --- | --- | --- | --- | --- | --- | --- |
| Hospitalization in a veterinary clinic | 3 (22) | 42 (759) | 826 | | 0.156 | | 2.46 | | 0.71-8.56 |
| Boarding kennel | 2 (23) | 42 (773) | 840 | | 0.533 | | 1.60 | | 0.37-7.02 |
| Animal shelter | 0 (25) | 12 (803) | 840 | | . | | . | | . |
| Kindergarten/  school | 0 (25) | 10 (805) | 840 | | . | | . | | . |
| Dog-/cat-show | 6 (19) | 154 (661) | 840 | | 0.524 | | 1.36 | | 0.53-3.45 |
| Health care/ rehabilitation facility | 0 (25) | 19 (796) | 840 | | . | | . | | . |
| In use as therapy dog | 0 (25) | 15 (800) | 840 | | . | | . | | . |
| Pet obedience school | 1 (24) | 134 (681) | 840 | | 0.130 | | 0.21 | | 0.03-1.58 |
| **Contacts – human/ animal** | | | | | | | | | |
| Owner of the tested pet has suffered from diarrhea during the last 4 weeks | 6 (14) | 86 (399) | 505 | | 0.171 | | 1.99 | | 0.74-5.32 |
| Person with chronic disease lives in the household | 9 (9) | 135 (332) | 485 | | 0.062 | | 2.46 | | 0.96-6.33 |
| Owner of the tested pet suffers from chronic disease | 10 (9) | 113 (370) | 502 | | 0.006 | | 3.64 | | 1.44-9.17 |
| Person with a previous positive *C. difficile* test lives in the household | 1 (8) | 12 (380) | 401 | | 0.211 | | 3.96 | | 0.46-34.21 |
| Owner of the tested pet was previously tested positive for  *C. difficile* | 0 (16) | 1 (437) | 454 | | . | | . | | . |
| **Contact to a hospitalized human or animal during the last 12 months** | | | | | | | | | |
| Human or animal | 10 (13) | 257 (457) | 737 | | 0.464 | | 1.37 | | 0.59-3.16 |
| Human | 6 (17) | 187 (527) | 737 | | 0.991 | | 0.99 | | 0.39-2.56 |
| Animal | 4 (19) | 98 (616) | 737 | | 0.617 | | 1.32 | | 0.44-3.97 |
| **Contact to a human or animal with the onset of diarrhea during the last 12 months** | | | | | | | | | |
| Human or animal | 13 (4) | 358 (251) | 626 | | 0.154 | | 2.28 | | 0.73-7.07 |
| Human | 11 (6) | 214 (379) | 610 | | 0.022 | | 3.25 | | 1.18-8.90 |
| Animal | 6 (11) | 213 (379) | 609 | | 0.954 | | 0.97 | | 0.35-2.66 |
| **Intensity of contact between participating dog/cat and its owner** | | | | | | | | | |
| The animal is allowed to… | | | |  | |  | |  | |
| … lie on the couch | 22 (3) | 671 (139) | 835 | | 0.502 | | 1.52 | | 0.45-5.15 |
| … sleep in bed | 18 (7) | 521 (285) | 831 | | 0.450 | | 1.41 | | 0.58-3.41 |
| … be washed in the tub/shower | 16 (9) | 352 (443) | 820 | | 0.057 | | 2.24 | | 0.98-5.12 |
| … be petted | 25 (0) | 808 (1) | 834 | | . | | . | | . |
| … feed out of the hand | 24 (1) | 749 (43) | 817 | | 0.756 | | 1.38 | | 0.18-10.43 |
| … lick the face | 18 (7) | 471 (319) | 815 | | 0.219 | | 1.74 | | 0.72-4.22 |
| other contacts | 9 (3) | 148 (165) | 325 | | 0.074 | | 3.34 | | 0.89-12.59 |
| **Contact between animals** | | | | | | | | | |
| **Contact between participating dog/cat and other animals** | | | | | | | | | |
| Contact | 20 (4) | 720 (88) | 832 | | 0.379 | | 0.61 | | 0.20-1.83 |
| Dogs | 13 (11) | 505 (302) | 831 | | 0.404 | | 0.71 | | 0.31-1.60 |
| Cats | 13 (11) | 458 (349) | 831 | | 0.801 | | 0.90 | | 0.40-2.03 |
| Sheep | 0 (24) | 17 (790) | 831 | | . | | . | | . |
| Poultry | 1 (23) | 74 (733) | 831 | | 0.413 | | 0.43 | | 0.06-3.23 |
| Wild animals | 0 (24) | 33 (774) | 831 | | . | | . | | . |
| Small companion animals | 2 (22) | 52 (755) | 831 | | 0.712 | | 1.32 | | 0.30-5.77 |
| Horses | 1 (23) | 73 (734) | 831 | | 0.421 | | 0.44 | | 0.06-3.28 |
| Cattle | 0 (24) | 14 (793) | 831 | | . | | . | | . |
| Pigs | 0 (24) | 4 (803) | 831 | | . | | . | | . |
| Others | 1 (23) | 47 (760) | 831 | | 0.733 | | 0.70 | | 0.09-5.32 |
| **Regular contact to infant animals** | | | | | | | | | |
| Contact | 8 (17) | 349 (449) | 823 | | 0.248 | | 0.61 | | 0.26-1.42 |
| Dogs | 4 (21) | 192 (606) | 823 | | 0.356 | | 0.60 | | 0.20- 1.77 |
| Cats | 4 (21) | 172 (626) | 823 | | 0.507 | | 0.69 | | 0.23- 2.05 |
| Sheep | 0 (25) | 8 (790) | 823 | | . | | . | | . |
| Poultry | 1 (24) | 8 (790) | 823 | | 0.191 | | 4.11 | | 0.49- 34.21 |
| Wild animals | 0 (25) | 2 (796) | 823 | | . | | . | | . |
| Small companion animals | 1 (24) | 9 (789) | 823 | | 0.228 | | 3.65 | | 0.44- 29.99 |
| Horses | 0 (25) | 4 (794) | 823 | | . | | . | | . |
| Cattle | 0 (25) | 4 (794) | 823 | | . | | . | | . |
| Pigs | 0 (25) | 1 (797) | 823 | | . | | . | | . |
| Others | 0 (25) | 3 (795) | 823 | | . | | . | | . |

CD: *Clostridium difficile* isolation; Ref.: reference category; OR: odds ratio; CI: confidence interval. *Authors’ comment*: bracketed data indicate the number of participants not applying to the variable in row.
