# Supplementary material for: The zoonotic potential of Clostridium difficile from small companion animals and their owners
Source: PLoS One. 2018 Feb 23;13(2):e0193411. doi: 10.1371/journal.pone.0193411 (PMC5825086; doi:10.1371/journal.pone.0193411)
Supplement: S3 Table — (DOCX) [file pone.0193411.s005.docx]

**Supplementary Table 3.** Complete univariate analysis for fecal shedding of *C. difficile* in animal owners.

|  | CD positive | CD negative | n | *p*-Value | OR | 95% CI |
| --- | --- | --- | --- | --- | --- | --- |
| **Demographic factors** | |  |  |  |  |  |
| **Age in years** |  |  | 566 |  |  |  |
| <1 | 2 | 1 | 3 | 0.001 | 70.00 | 5.41-905.30 |
| 1-4 | 1 | 4 | 5 | 0.072 | 8.75 | 0.82-93.11 |
| 5-17 | 0 | 26 | 26 | . | . | . |
| 18-44 | 5 | 175 | 180 | Ref. |  |  |
| 45-64 | 7 | 274 | 281 | 0.850 | 0.89 | 0.28-2.86 |
| 65-87 | 2 | 69 | 71 | 0.986 | 1.01 | 0.19-5.35 |
| **Sex** | | | | | | |
| Female (Male) | 10 (7) | 384 (171) | 572 | 0.367 | 0.64 | 0.24-1.70 |
| **Place of residence** | |  |  |  |  |  |
| District of Germany | (no significance regarding a certain district of Germany could be distinguished) | | | | | |
| Countryside (large/provincial city) | 11 (6) | 316 (230) | 563 | 0.575 | 1.33 | 0.49- 3.66 |
| **Profession/ field of occupation** | | | | | | |
| Agriculture | 1 (16) | 21 (540) | 578 | 0.653 | 1.61 | 0.20- 12.70 |
| Food production | 0 (17) | 5 (556) | 578 | . | . | . |
| Health care | 1 (16) | 101 (460) | 578 | 0.225 | 0.28 | 0.04-2.17 |
| Other field of action | 4 (13) | 293 (268) | 578 | 0.028 | 0.28 | 0.09-0.87 |
| No current occupation (e.g. retirement, parental leave) | 9 (8) | 138 (423) | 578 | 0.013 | 3.45 | 1.31-9.11 |
| Not specified | 15 (2) | 519 (42) | 578 | 0.516 | 0.61 | 0.13- 2.74 |
| **Health status** | | | | | | |
| Diarrhea during the last 4 weeks | 3 (14) | 106 (451) | 574 | 0.886 | 0.91 | 0.26-3.23 |
| Chronic disease | 6 (10) | 134 (423) | 573 | 0.224 | 1.89 | 0.68-5.31 |
| Chemotherapy during the last 12 months | 0 (17) | 3 (555) | 575 | . | . | . |
| Previous positive test for *C. difficile* | 0 (14) | 2 (500) | 516 | . | . | . |
| Hospitalization for at least 1 week during the last 12 months | 2 (15) | 45 (506) | 568 | 0.598 | 1.50 | 0.33-6.76 |
| **Medication** | | | | | | |
| Anti-inflammatory drugs | 3 (14) | 81 (475) | 573 | 0.724 | 1.26 | 0.35-4.47 |
| Proton pump inhibitors | 2 (15) | 49 (505) | 571 | 0.679 | 1.37 | 0.31-6.18 |
| Antibiotics | 9 (8) | 70 (485) | 572 | <0.001 | 7.79 | 2.91-20.87 |

| **Food consumption** | | | | | | |
| --- | --- | --- | --- | --- | --- | --- |
| Tap water as cold drink | 14 (3) | 453 (83) | 553 | 0.809 | 0.86 | 0.24-3.04 |
| Raw milk/-products | 12 (4) | 394 (141) | 551 | 0.903 | 1.07 | 0.34-3.38 |
| Raw meat/-products | 14 (3) | 389 (146) | 552 | 0.384 | 1.75 | 0.50-6.18 |
| Ready-to-eat-salads | 11 (6) | 372 (167) | 556 | 0.706 | 0.82 | 0.30-2.26 |
| Probiotics | 7 (10) | 292 (250) | 559 | 0.306 | 0.60 | 0.22-1.60 |
| **Contacts – human/ animal** | | | | | | |
| Children younger than 16 years live in the same household | 3 (13) | 120 (433) | 569 | 0.778 | 0.83 | 0.23- 2.97 |
| Person with chronic disease lives in the household | 6 (10) | 160 (378) | 554 | 0.506 | 1.42 | 0.51-3.97 |
| Person or animal with a previous positive *C. difficile* test lives in the household | 0 (13) | 15 (426) | 454 | . | . | . |
| **Contact to a hospitalized human or animal during the last 12 months** | | | | | | |
| Human or animal | 5 (11) | 232 (233) | 481 | 0.152 | 0.46 | 0.16-1.33 |
| Human | 4 (1) | 195 (38) | 238 | 0.826 | 0.78 | 0.08-7.17 |
| Animal | 2 (3) | 60 (174) | 239 | 0.476 | 1.93 | 0.32-11.85 |
| **Contact to a human or animal with the onset of diarrhea during the last 12 months** | | | | | | |
| Human or animal | 8 (6) | 283 (144) | 441 | 0.480 | 0.68 | 0.23-1.99 |
| Human | 4 (10) | 163 (264) | 441 | 0.469 | 0.65 | 0.20-2.10 |
| Animal | 7 (7) | 177 (250) | 441 | 0.525 | 1.41 | 0.49-4.10 |
| **Animal husbandry** (additionally to tested dog/cat) | | | | | | |
| Keeping farm or companion animals | 9 (8) | 342 (219) | 578 | 0.506 | 0.72 | 0.27-1.90 |
| Dogs | 4 (13) | 163 (398) | 578 | 0.622 | 0.75 | 0.24-2.34 |
| Cats | 6 (11) | 199 (362) | 578 | 0.988 | 0.99 | 0.36-2.72 |
| Sheep | 1 (16) | 10 (551) | 578 | 0.252 | 3.44 | 0.42-28.54 |
| Poultry | 3 (14) | 40 (521) | 578 | 0.118 | 2.79 | 0.77-10.12 |
| Wild animals | 0 (17) | 2 (559) | 578 | . | . | . |
| Small companion animals | 1 (16) | 38 (523) | 578 | 0.885 | 0.86 | 0.11-6.66 |
| Horses | 0 (17) | 50 (511) | 578 | . | . | . |
| Cattle | 1 (16) | 9 (552) | 578 | 0.215 | 3.83 | 0.46-32.09 |
| Pigs | 0 (17) | 5 (556) | 578 | . | . | . |
| Others | 1 (16) | 56 (505) | 578 | 0.582 | 0.56 | 0.07-4.33 |

| **Multiple contacts to other animals not in care by participant** | | | | | | | | | |
| --- | --- | --- | --- | --- | --- | --- | --- | --- | --- |
| Contact | 12 (5) | 417 (144) | 578 | | 0.728 | | 0.83 | | 0.29-2.39 |
| Dogs | 12 (3) | 378 (105) | 498 | | 0.872 | | 1.11 | | 0.31-4.01 |
| Cats | 8 (7) | 270 (213) | 498 | | 0.844 | | 0.90 | | 0.32-2.53 |
| Sheep | 1 (14) | 40 (443) | 498 | | 0.823 | | 0.79 | | 0.10-6.17 |
| Poultry | 3 (12) | 46 (437) | 498 | | 0.193 | | 2.38 | | 0.65-8.72 |
| Wild animals | 1 (14) | 38 (445) | 498 | | 0.865 | | 0.84 | | 0.11-6.53 |
| Small companion animals | 2 (13) | 75 (408) | 498 | | 0.817 | | 0.84 | | 0.19-3.78 |
| Horses | 2 (13) | 127 (356) | 498 | | 0.273 | | 0.43 | | 0.10-1.94 |
| Cattle | 2 (13) | 52 (431) | 498 | | 0.753 | | 1.28 | | 0.28-5.81 |
| Pigs | 1 (14) | 40 (443) | 498 | | 0.823 | | 0.79 | | 0.10-6.17 |
| Others | 1 (14) | 38 (445) | 498 | | 0.865 | | 0.84 | | 0.11-6.53 |
| **Intensity of contact between participating animal and its owner** | | | | | | | | | |
| The animal is allowed to… | | | |  | |  | |  | |
| … lie on the couch | 12 (3) | 389 (99) | 503 | | 0.978 | | 1.02 | | 0.28-3.68 |
| … sleep in bed | 9 (6) | 298 (186) | 499 | | 0.902 | | 0.94 | | 0.33-2.67 |
| … be washed in the tub/shower | 8 (7) | 209 (269) | 493 | | 0.463 | | 1.47 | | 0.52-4.12 |
| … be petted | 15 (0) | 488 (1) | 504 | | . | | . | | . |
| … feed out of the hand | 14 (1) | 443 (32) | 490 | | 0.991 | | 1.01 | | 0.13-7.94 |
| … lick the face | 9 (6) | 268 (206) | 489 | | 0.790 | | 1.15 | | 0.40-3.29 |
| other contacts | 5 (4) | 84 (133) | 226 | | 0.319 | | 1.98 | | 0.52-7.58 |

CD: *Clostridium difficile* isolation; Ref.: reference category; OR: odds ratio; CI: confidence interval. *Authors’ comment*: bracketed data indicate the number of participants not applying to the variable in row.
